# Heterochronic development of lateral plates in the three-spined stickleback induced by thyroid hormone level alterations

**DOI:** 10.1371/journal.pone.0194040

**Published:** 2018-03-09

**Authors:** Aleksey A. Bolotovskiy, Marina A. Levina, Jacquelin DeFaveri, Juha Merilä, Boris A. Levin

**Affiliations:** 1 Papanin Institute for Biology of Inland Waters, Russian Academy of Sciences, Borok, Yaroslavl Prov., Russia; 2 Ecological Genetics Research Unit, Department of Biosciences, University of Helsinki, Helsinki, Finland; 3 Cherepovets State University, Cherepovets, Russia; Universiteit Gent, BELGIUM

## Abstract

The three-spined stickleback *Gasterosteus aculeatus* is an important model for studying microevolution and parallel adaptation to freshwater environments. Marine and freshwater forms differ markedly in their phenotype, especially in the number of lateral plates, which are serially repeated elements of the exoskeleton. In fishes, thyroid hormones are involved in adaptation to salinity, as well as the developmental regulation of serially repeated elements. To study how thyroid hormones influence lateral plate development, we manipulated levels of triiodothyronine and thiourea during early ontogeny in a marine and freshwater population with complete and low plate phenotypes, respectively. The development of lateral plates along the body and keel was heterochronic among experimental groups. Fish with a low dosage of exogenous triiodothyronine and those treated with thiourea exhibited retarded development of bony plates compared to both control fish and those treated with higher a triiodothyronine dosage. Several triiodothyronine-treated individuals of the marine form expressed the partial lateral plate phenotype. Some individuals with delayed development of lateral plates manifested 1–2 extra bony plates located above the main row of lateral plates.

## Introduction

The three-spined stickleback *Gasterosteus aculeatus* L. is a circumpolar species distributed across the Northern hemisphere, and it has emerged as an important model organism for studying many contemporary problems in ecology and evolution [1–3]. The species is phenotypically very diverse, but there are two basic forms recognized as marine and freshwater ecotypes [4]. The marine form (MF) is ancestral, and its morphology is thought to be conserved since the Miocene [5, 6]. Multiple colonizations of freshwater habitats have resulted in significant phenotypic changes [7, 8]. One of the most striking morphological differences between the two main forms is in the number of bony lateral plates protecting the body. These bony plates, as well as fin rays, are part of the exoskeleton, which is largely independent from the endoskeleton in terms of function, development and evolution [9–11]. The marine form (MF) of the three-spined stickleback has numerous (30–36) lateral bony plates covering most or all of the body, while the freshwater form (FF) usually has less than 10 (typically 5–7) lateral plates along the anterior part of the body [12]. An intermediate partially plated form occurs mainly in hybrids between low and fully plated phenotypes, but also as an independently evolved form in some populations (e.g. [13, 14]).

Apart from plate numbers, there are also many other phenotypic differences between marine and freshwater forms [15–17], some of which have a genetic basis that has been identified [18–21]. The fast evolution of the MF to FF occurs via rapid assembly of freshwater genotypes from standing genetic variation in the ancestral MF, driven by strong selection acting simultaneously on multiple loci favoring life in freshwater [22–24]. However, environmental conditions have also been shown to affect the expression of traits [15, 25]. An important mediator of how genes respond to the environment is hormones [26]. In particular, thyroid hormones (THs) are known to regulate development of morphological, physiological, biochemical, and behavioral traits of many fish species, including three-spined sticklebacks [27–31]. Specifically, THs signaling pathway is involved in complex adaptations to the freshwater environment in stickleback [31]. For example, the FF exhibits a lower plasma concentration of thyroid hormone (thyroxine), as well as a lower metabolic rate, the latter of which is likely adaptive for permanent residency in small water bodies. Kitano et al. [31] suggested that divergence in hormone concentrations underlay evolutionary changes in the coordinated physiological and behavioral traits that comprise an organism's integrated phenotype.

Several recent studies have demonstrated the strong influence of THs on fish phenotype, including skeletal characters. Serially repeated elements (SREs), such as the number of lateral line scales, fin rays, and pharyngeal teeth, have been shown to change significantly in experiments with thyroid status regulation in cyprinid fishes [32–36]. In these studies, adding exogenous hormones during early development resulted in an accelerated developmental rate and fewer numbers of SREs (oligomerization), while exposure to goitrogen caused delayed development and increased SRE numbers (polymerization).

The aim of the present study was to examine the influence of thyroid hormones on the development of meristic traits, with special attention towards the lateral bony plates in the marine and freshwater forms of the three-spined stickleback. In particular, we were interested in whether the developmental and phenotypic responses of stickleback to hormonal treatments were similar to those previously observed in cyprinids.

## Results

### T_3_ concentrations in wild and experimental fish

**Fig 1:**
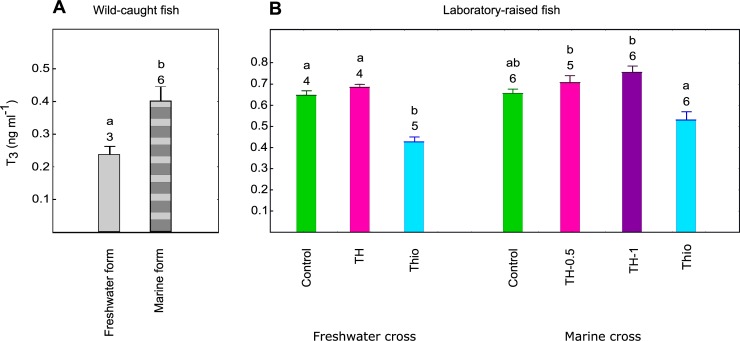
T_3_ concentrations in the three-spined stickleback. (A) Plasma T_3_ concentrations of wild caught three-spined sticklebacks. (B) Whole-body T_3_ concentrations in laboratory-raised three-spined sticklebacks (61 dpf). Middle point is median, box and whisker are quartiles and range respectively. Samples are combined and each contains between 4 and 13 individuals; the sample size for each group is indicated above the bar. Different lowercase letters above the bars indicate significant differences between forms (p < 0.05, Mann-Whitney U-test for wild fish) or between experimental groups (p < 0.05, Kruskal-Wallis test with Dunn’s *post hoc* test for laboratory-raised fish). Differences between groups were tested independently within each panel and each cross.

Among the wild fish, plasma T_3_ concentrations differed significantly between the MF and FF groups (Mann-Whitney U-test, U = 1.000, df = 8, p = 0.039), with the MF fish having concentrations twice as high as the FF fish (Median = 0.40 ng/ml and 0.20 ng/ml, respectively; Fig 1A).

Whole body T_3_ concentrations differed between some of the experimental groups. The concentrations were significantly higher in TH-fish compared to the Thio-treated fish in both FF and MF crosses (Kruskal-Wallis tests, p < 0.05, Fig 1B). T_3_ concentration in TH-fish was slightly higher, though not significantly, than the control group in both MF and FF crosses (Fig 1B). A dose-dependent trend towards higher T_3_ concentration between the two TH groups in the MF cross was noted. There was no difference in T_3_ concentration between the Thio and control fish in the MF crosses, but the Thio-fish in the FF crosses had significantly lower T_3_ concentrations compared to the controls (Fig 1B).

### Experimental effects on meristic characters

**Fig 2:**
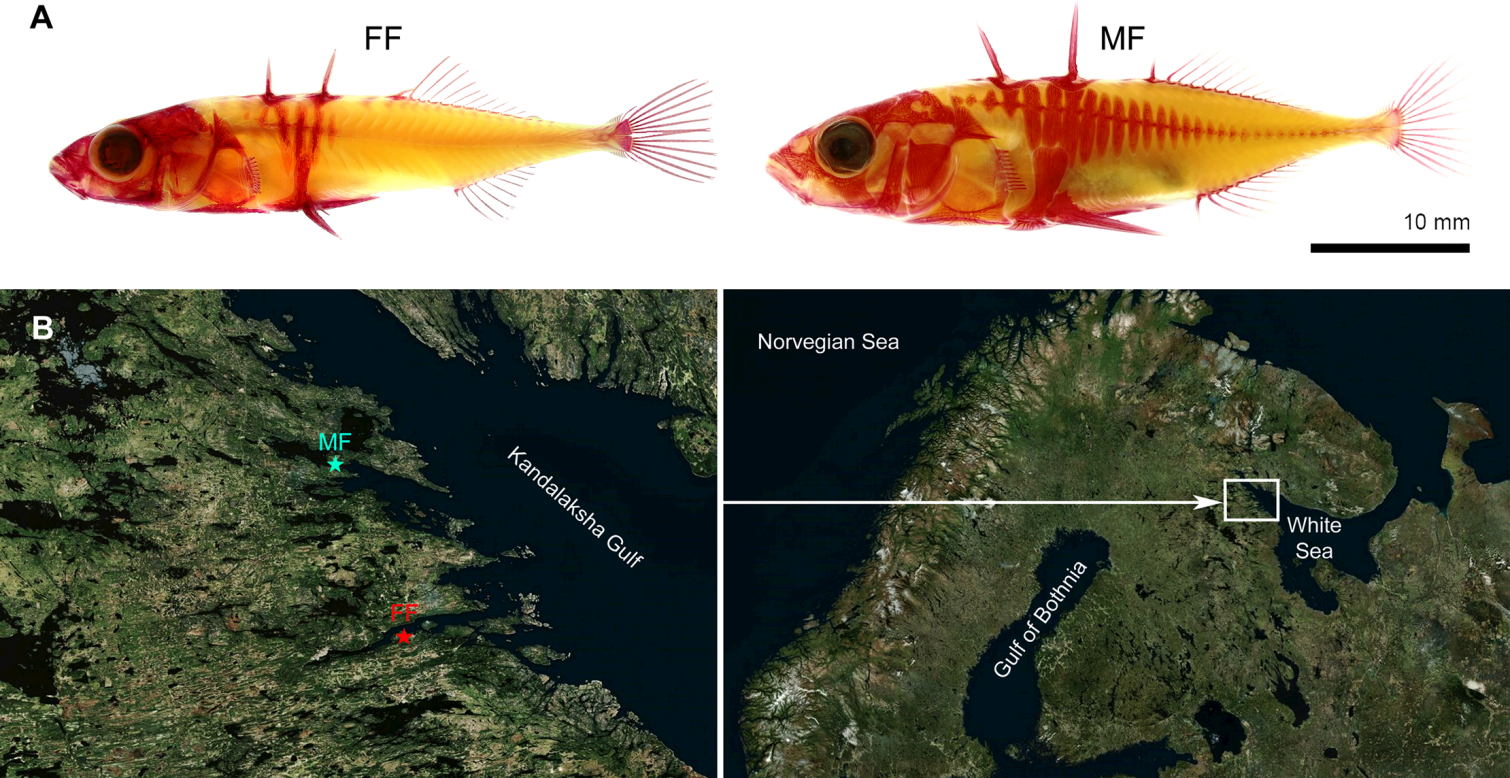
(A) Alizarin red stained control fish of the freshwater (FF, 130 dpf age) and marine (MF, 101 dpf) forms. (B) Sites of sampling of parental fish at the White Sea basin. Maps were obtained from http://www.sasgis.org.

All non-treated fish (controls) of the MF and FF crosses demonstrated either complete or low-plated phenotypes, respectively (Fig 2A). Control fish from the MF and FF crosses differed significantly in LP (MF > FF), Va (MF > FF), Vc (FF > MF) and C (FF > MF) (Mann-Whitney, p < 0.05; Table 1). Among the MF treated groups, Thio-fish had significantly more LPs than TH-0.5 fish (Kruskal-Wallis, p < 0.05, Table 1). Moreover, these groups are significantly different, even upon exclusion of the TH-0.5 individuals with a partial lateral plate phenotype (see below).

**Table 1:** Mean (±SEM; range) values of meristic traits in different experimental groups separately for FF and MF fish.

	Freshwater form (FF)			Marine form (MF)			
	Thio (n = 13)	Control (n = 24)	TH (n = 16)	Thio (n = 19)	Control (n = 14)	TH-0.5 (n = 43)	TH-1 (n = 11)
D	10.85±0.15 10–12	11.42±0.13 10–13	11.31±0.12 11–12	11.21±0.24 9–13	11.57±0.25 10–13	11.53±0.09 11–13	11.73±0.14 11–12
A	8.62±0.14 8–9	8.63±0.13 8–10	8.50±0.16 8–10	8.32±0.13 7–9	8.36±0.17 7–9	8.65±0.10 7–10	8.45±0.21 7–9
P	10.00±0.00 10	9.96±0.04 9–10	9.94±0.06 9–10	10.00±0.00 10	10.00±0.00 10	9.98±0.04 8–11	10.09±0.09 10–11
C	25.15±0.30 24–27	24.63±0.23 22–26	25.38±0.31 24–28	23.84±0.43 20–27	23.29±0.29 22–26	22.93±0.24 19–26	23.45±0.41 20–25
LP	5.85±0.21 4–7	5.90±0.19 4–8	5.47±0.29 4–9	**33.13±0.24**^**a**^ **30–36**	**32.57±0.27**^**ab**^ **31–36**	**31.69±0.31**^**b**^ **21–35**	**32.64±0.21**^**ab**^ **32–35**
GR	20.62±0.21 19–23	20.92±0.19 18–23	21.03±0.21 19–23	21.08±0.23 19–24	20.73±0.28 19–23	20.58±0.17 16–23	21.14±0.19 20–22
Vt	31.54±0.14 31–32	31.54±0.10 31–32	31.31±0.12 31–32	31.58±0.18 30–33	31.50±0.14 31–32	31.33±0.11 30–32	31.09±0.21 30–32
Va	14.46±0.18 14–16	14.08±0.06 14–15	14.06±0.06 14–15	14.74±0.13 14–16	14.69±0.17 14–16	14.74±0.07 14–15	14.73±0.14 14–15
Vc	17.00±0.20 16–18	17.46±0.12 16–18	17.25±0.14 16–18	16.84±0.21 15–18	16.77±0.20 16–18	16.58±0.11 15–18	16.36±0.24 15–17

For trait abbreviations, see Methods. Significant differences were observed between experimental groups in LP for MF fish (indicated with different lowercase letters, Kruskall-Wallis test with Dunn’s *post hoc* test, p < 0.05).

**Fig 3:**
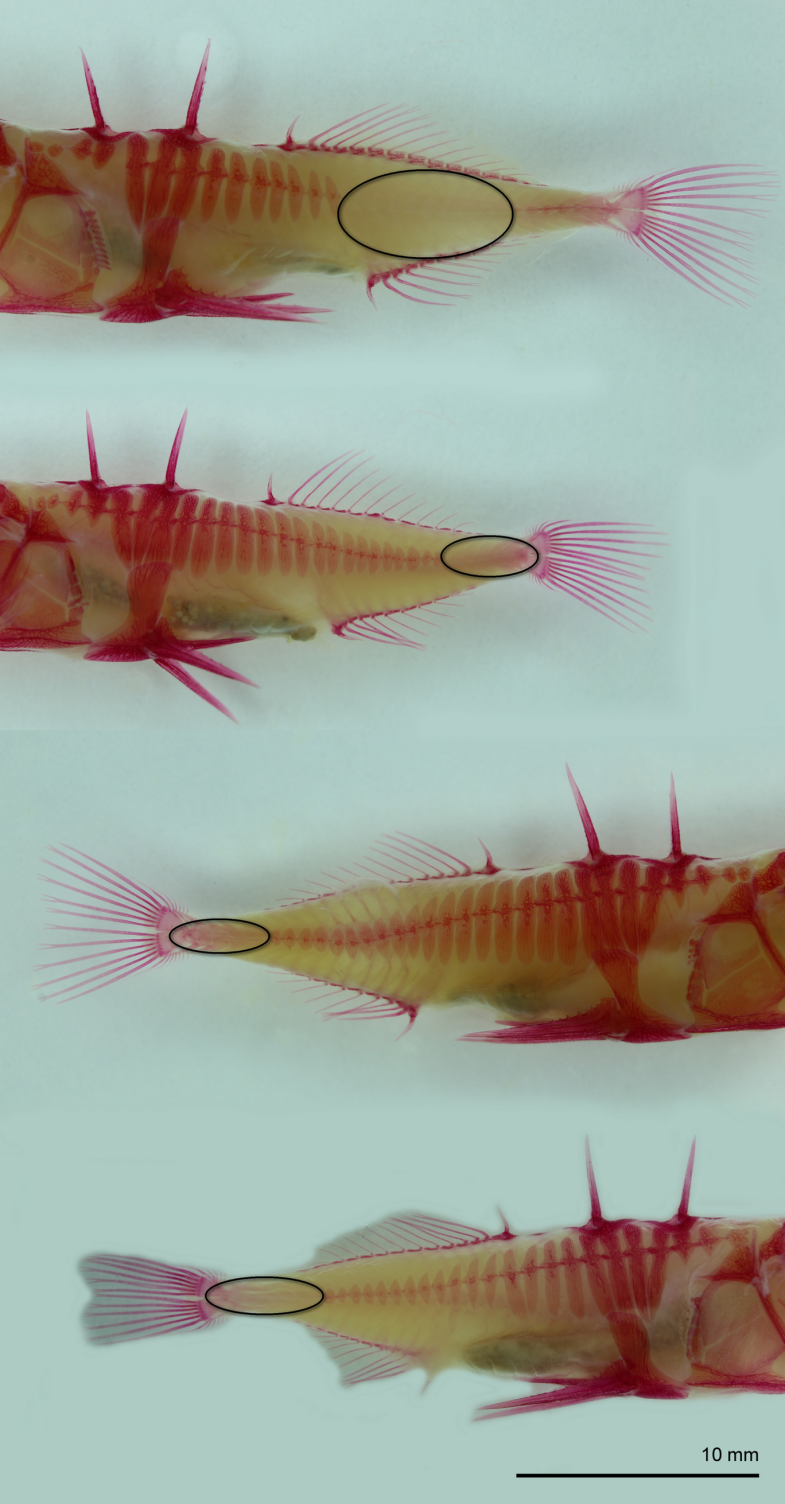
Four of the TH-0.5 treated individuals (101 dpf) of the MF with the partial lateral plate phenotype. Ellipses delineate areas with no lateral plates.

Four of the TH-0.5 treated individuals of the MF cross lacked several lateral plates on one (n = 3) or both sides (n = 1) of the body (Fig 3). Apart from lateral plates, there were no significant differences in mean values of all other meristic traits between treatment groups, except for a non-significant tendency for the control fish to have higher D count as compared to the Thio-fish in the FF crosses (Kruskall-Wallis, p = 0.07).

### Heterochronies in LP development in the marine form

**Fig 4:**
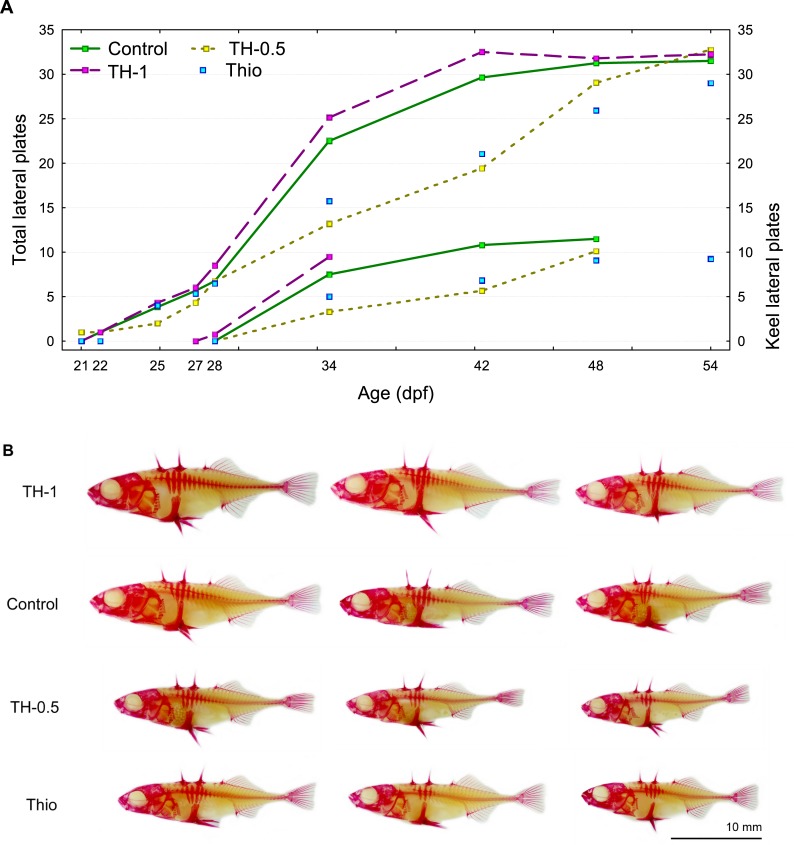
(A) Heterochronies in development (appearance) of lateral (left y-axis, four upper lines) and keel plate (right y-axis, four lower lines) in different experimental groups of the MF fish. Development of keel plates is given until forming a single continuous row with anterior plates. Plotted values are treatment-specific means. (B) Differences in degree of development of the lateral plates in MF cross at 42 dpf. Representative specimens of three sizes (largest, intermediate, and smallest) are shown from each experimental group.

Fish from the different experimental groups demonstrated heterochronic development of LP number (Fig 4A and 4B). The first anterior plates (three plates) appeared in the control, TH-0.5, and TH-1 groups almost at the same age (21–22 dpf). Thio-treated fish had no plates at 22 dpf; the first anterior plates in the Thio-fish appeared by 25 dpf (Fig 4A).

The keel plates appeared first in the TH-1 fish (28 dpf), and keel plates in the control, TH-0.5 and Thio-fish appeared between 29–34 dpf (Fig 4A).

The fully plated phenotype (*i*.*e*. anterior and keel plates form a single continuous row, as seen in the typical adult marine form) was attained earliest (42 dpf) in the TH-1 individuals (Fig 4B). By 48 dpf, full plate development was complete in most of the control and TH-0.5 fish, whereas Thio-treated fish demonstrated suppression of LP development; half of the individuals took 54 dpf to express the fully plated phenotype (Fig 4A).

In contrast to lateral plates, all other meristic characters did not demonstrate obvious developmental shifts in response to either of the hormone treatments.

### Induction of an additional row of bony plates by thyroid regulation

**Fig 5:**
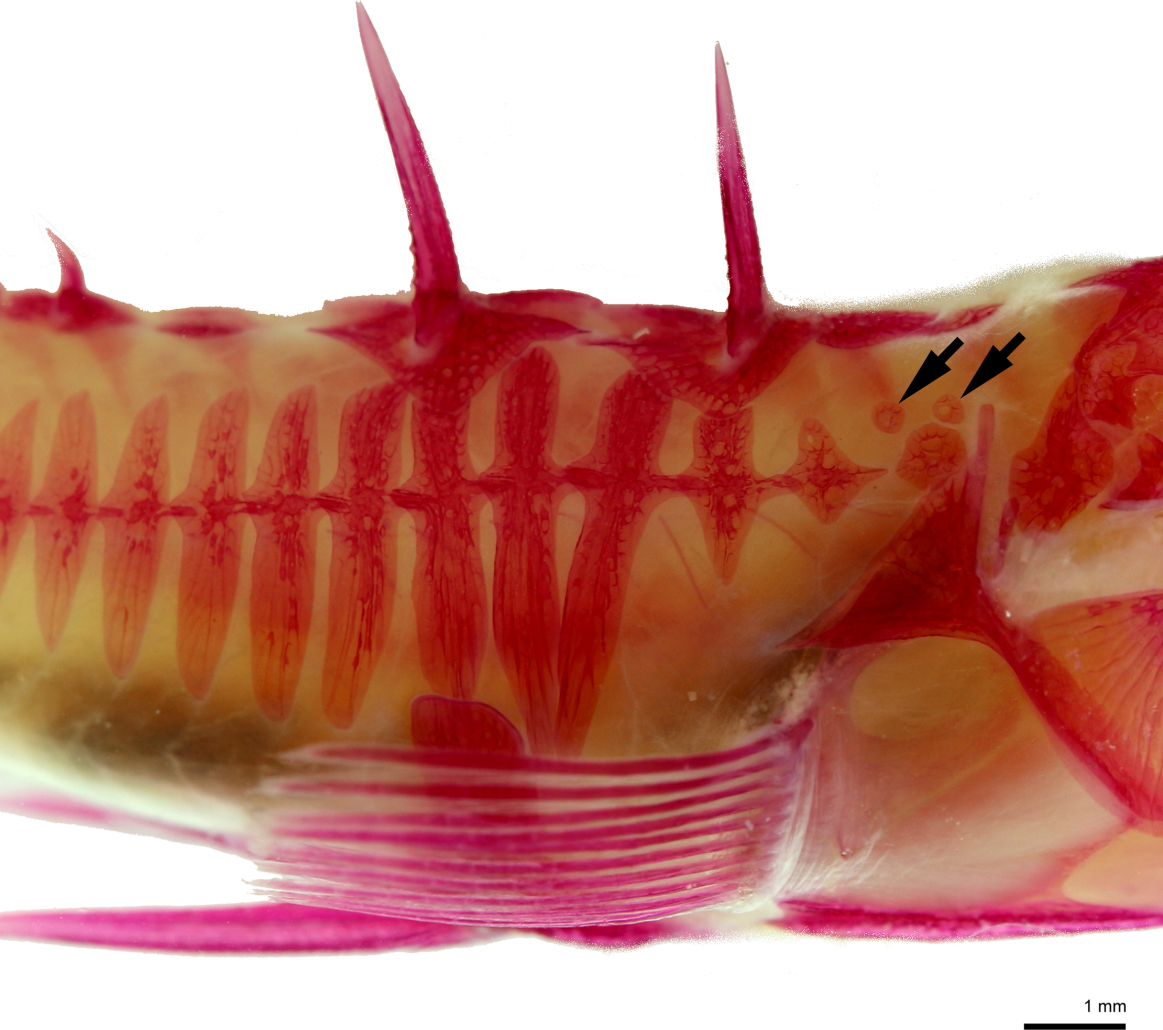
Additional two small bony plates (arrows). Additional plates are located above the main row of lateral plates, behind the head as exemplified from a TH-0.5 fish of marine form (fully plated phenotype, right side), age 101 dpf, TL = 34.7 mm (alizarin red S stained). Scale bar = 1 mm.

Seven of the treated individuals with a fully plated phenotype (MF) developed an additional small-sized bony plate(s) on the body, behind the head and above the main row of lateral plates (Fig 5). This extra plating was detected in four individuals from the TH-0.5 group and three individuals from the Thio group.

## Discussion

When experienced early in ontogeny, experimental changes in thyroid hormone concentrations can result in developmental heterochronies of skeletal structures in fishes [27, 32, 34, 37]. In general, hormonally-induced heterochronies frequently affect the number of serially repeated skeletal elements like scales, fin rays and pharyngeal teeth [36, 38, 39]. Specifically, thyroid treatment usually induces accelerated development of calcification and squamation, which often translates into reduced numbers of skeletal elements and scales, whereas thyroid disruptors tend to delay development and can lead to increased numbers of skeletal elements [27, 32, 34, 37].

In our experiment, thyroid treatment did not have a significant effect on whole body T_3_ concentration. Moreover, among our lab-reared samples, T_3_ levels were similar between marine and freshwater offspring, both in the control and TH treatments. An earlier study of lab-reared stickleback reported contrasting results to ours–lab-reared anadromous stickleback had higher levels of T_3_ than resident freshwater individuals [31]. However, it is important to note the difference in age/developmental stage of the samples used in both studies. Specifically, the lab-reared stickleback in the previous study were sampled after 8–12 months of treatment and likely closer to reproductive condition, whereas our study commenced after 3 months when the fish were still juveniles. Hence, considering the correlation between thyroid status and reproductive status [40], it is possible that we did not observe differences in T_3_ levels between experimental fish because they were not in reproductive condition. Alternatively, the discrepancy between our results could be methodological–T_3_ concentration in the Kitano et al. [31] study was measured directly from plasma, whereas whole bodies were analyzed in the juveniles in our study. The same is true for the wild fish in our study, in which T_3_ levels were higher in the marine form, as determined from plasma samples. Hence, it is possible that T_3_ levels do differ between marine and freshwater lab-reared fish in our study, but these differences were diffused by other substances and lipids in the tissue that might have interfered with the assay. In fact, earlier stickleback studies measuring T_3_ in response to perchlorate exposure have also failed to detect differences in thyroid levels between treatment groups, in spite of significant morphological developmental differences [41, 42]. There too, T_3_ concentration was measured using whole-bodies, possibly suggesting that the use of whole bodies is not sensitive enough to detect changes in T_3_ levels in stickleback.

Regardless of the lack of detectable T_3_ differences between TH-treated and control lab-reared fish, thyroid treatment nevertheless had a significant effect on the development of lateral plate number. As expected, the TH-treated fish demonstrated an accelerated developmental rate of lateral plates. The final number of plates did not differ from those in the other groups, however there was a tendency for the TH-0.5-treated fish to express the partially plated phenotype (*i*.*e*. fewer plates). On the other hand, exposure to thiourea significantly retarded the development of lateral plates–the full range of LPs was registered later than in the other treatment groups. This prolonged developmental period resulted in an increased total number of lateral plates as compared with other groups (although this was only significant when compared with the TH-0.5 fish).

Interestingly, this heterochronic development only affected the marine form, most notably in the development of the keel and/or the posterior non-structural plates. Plate development is known to be largely controlled by the *ectodysplasin A* (*Eda*) gene, where different mutations lead to the expression of the different plate morphs [22]. Accordingly, most of the fully plated marine samples in our study had divergent alleles at the *Eda* locus compared to the low plated freshwater samples. In light of the fact that thyroid hormones can suppress or activate expression of many genes–including those involved in ossification, skeletal development and lateral line/seismosensory organs in fishes [43, 44]–it is possible that thyroid hormones also regulate the *Eda* gene in stickleback. Moreover, since the different forms/plate morphs responded differently to the thyroid treatments (*i*.*e*. heterochronic development only observed in the marine form), it is possible that thyroid hormones are regulated differently by the different *Eda* genotypes. In fact, an earlier study has demonstrated divergence in thyroid hormone physiology between marine (fully plated) and freshwater (low plated) stickleback, where both thyroid hormone levels and mRNA levels in thyroid stimulating hormone (*TSH*β*2*) were significantly higher in marine populations [31]. In addition, only the marine form displayed changes in expression of *TSH*β*2* in response to photoperiod, suggesting differences in thyroid hormone signaling pathways between forms [31].

Some individuals of the fully plated phenotype (marine form) manifested additional one or two small-sized bony plates on the body, posterior to the head and above the main row of lateral plates (Fig 5). This extra row of bony plates was observed in some of the experimental fish exposed to thiourea, which suppressed/delayed development of lateral bony plates. Previous experiments investigating the effect of thyroid regulation on developmental rate in cyprinid fishes show that artificially retarded development can result in increased (or polymerized) SRE numbers, and scales in particular [32, 39, 45, 46]. Hence, the appearance of extra plates in the thiourea-treated fish are in line with earlier findings from cyprinids, particularly since both the number of scales and number of anteroposterior rows of scales were increased in earlier studies as they were in ours. However, extra plates were also observed in some of the low dosage triiodothyronine-treated fish, which was unexpected. It is noteworthy that some of these fish did express an initial delayed development until 42 dpf (Fig 4). It is possible that this was a result of nonmonotonic (non-lineare) dose-response, where the effects of low doses cannot be predicted by the effects observed at high doses [47]. We nevertheless suggest that delayed lateral plate development in three-spined stickleback is achieved through thyroid regulation, which promoted the appearance of the phenotype with additional bony scutes on the body.

Although we are not aware of any other published examples of such additional bony plates in wild or lab-reared three-spined sticklebacks, we have noted the presence of additional plates in several wild sticklebacks from the Baltic Sea. Unlike the lab-reared individuals that expressed the extra plates above the main row, these wild caught specimens expressed extra plating below the main row of plates (S1 Fig). Interestingly, lateral plate morphology has been exhaustively screened throughout the entire Baltic Sea and connecting Danish straits [48]. However, the only sampling location where stickleback with additional plates were found was along the Russian coast of the Gulf of Finland. A recent survey of dioxin concentrations in the Baltic Sea revealed that sediments from this particular region consistently had the highest levels of dioxins [49], which are known endocrine disruptors with specific effects on thyroid metabolizing enzymes [50]. For example, exposure of dioxins has been shown to reduce the concentration of thyroid hormone [51], hence it is possible that the extra plating observed in the wild stickleback from the Gulf of Finland was a result of thyroid disruption induced by the elevated dioxins.

## Conclusions

Manipulation of thyroid level in developing sticklebacks resulted in heterochronies of development of lateral plates. Thiourea-treated fish had a significantly higher number of lateral plates compared to triiodothyronine-treated fish. Some sticklebacks with delayed development displayed an extra row of small-sized bony plates located above the main lateral plate row. Further studies are needed for understanding the mechanisms of thyroid effect on phenotypic features of sticklebacks, particularly those that are regulated by genes controlling ectodermal elements. In addition, thyroid hormones are a useful experimental tool for studying the mechanisms of loss and gain of serially repeated skeletal elements in fishes.

## Methods

All used methods were approved by the ethics committee of Papanin Institute for Biology of Inland Waters Russian Academy of Sciences (Borok, Yaroslavl Prov., Russia), and all experiments were performed in accordance with approved guidelines and regulations.

### Experimental design

Parents of the MF were caught in the Kandalaksha Bay of the White Sea near the Pertsov White Sea Biological Station of Lomonosov Moscow State University (WSBS MSU; 66°33’12"N, 33°06’17"E), Republic of Karelia, Russia. Parents of the FF were caught in an isolated freshwater lake, Mashinnoe (66°17'47"N, 33°22'03"E), Republic of Karelia, Russia (Fig 2B). Females of each form were stripped; eggs from different females within the same form (5 FF and 4 MF) were mixed in separate Petri dishes. Fish were killed with an overdose of anesthetic MS-222. Sperm was collected by macerating the testes of several males within each form (3 FF and 5 MF), and used to fertilize the mixed clutches of eggs from females of the same form. All parents were then fixed in 10% formalin and stained with Alizarin red S (Reachem, Russia); see below for details on staining protocol. Fertilized eggs were incubated in 5 L plastic containers using seawater from the White Sea or freshwater from a spring for incubation of marine and freshwater eggs, respectively. The progeny were kept at the following treatment regimes, which started just after fertilization (within 30 min) and continued until the end of experiment (101 dpf and 130 dpf for MF and FF, respectively): (i) alkaline solution of 3,5,3’-triiodothyronine T_3_ (Sigma Aldrich) at a concentration of 0.5 and 1 ng/ml for the MF, and 0.5 ng/ml for the FF (referred to as TH-fish); (ii) thiourea (Chimmed, Russia) solution, a goitrogen that blocks the synthesizing activity of the thyroid gland and usually leads to decreased endogenous TH-level (0.015% for both forms; referred to as Thio-fish); (iii) clean water (control). Previous studies on zebrafish *D*. *rerio* [52–54] and rainbow trout *Oncorhynchus mykiss* [55, 56] demonstrate that exogenous thyroid hormones are effectively absorbed by embryos and young fish from water (i.e. chorion is not a barrier for T_3_).

During the incubation period, 100% water changes were performed twice daily, and hormone/goitrogen were added to maintain the designated concentrations. Several days before hatching, experimental groups were transferred from the WSBS MSU to the laboratory in IBIW RAS, where seawater (18–20‰) was prepared by mixing sea salt (Tetra Marine SeaSalt, Germany) with distilled water. Water temperatures in all treatments was 10–11°C during the first days of egg incubation. After hatching, the larvae were transferred to 40 L tanks and temperature was gradually increased to 20–22°C. Tank bottoms were cleaned and 30% water changes were performed daily; designated concentrations of hormone/goitrogen were maintained through addition of each respective solution. The water in the tanks was gently aerated but not filtered. Larvae and juveniles were exposed to a 12L:12D photoperiod. Larvae were first fed with plankton food (Sera Micron, Germany) and *Artemia* nauplii. Frozen bloodworms and *Daphnia* were introduced to their diet at the juvenile stage, and provided *ad libitum*. The experimental and control fish were kept under identical conditions (*viz*. stocking density, temperature, oxygen conditions, etc.).

### Genotyping of parental fish

The parents were genotyped to verify that their lateral plate phenotype were concordant with their genotypes at the *Ectodisplasin A* (*Eda*) locus, known to contribute to lateral plate phenotype [22]. DNA was extracted from ethanol-preserved pectoral fin tissue using a standard salt method [57]. An indel polymorphism in intron 1 of the *Eda* gene (Stn382F 5’-CCC TTA GAG AAT TTC CTA GCA G-3’, and Stn382R 5’-CTT GTC CCG GAT CAT ACG C-3’ from Colosimo et al. [22]) was amplified, generating characteristic band sizes of 150 bp for the low-plated allele, and a 218 bp fragment for the fully plated allele [22]. PCR conditions followed the PFC8 protocol from Colosimo et al. [22], except that a total of 30 cycles of PCR and an annealing temperature of 56°C were used. PCR products were visualized on 2% agarose gels. All FF parents were homozygous for the low-plated allele, and most MF parents were homozygous for the completely plated allele, except for one male that was heterozygous (S2 Fig).

### Staining and morphological analyses

At each sampling point during development, three to eight individuals from each group were collected and preserved in 10% neutral-buffered formalin before further staining with alizarin red S [58]. Fish were cleared in 1% KOH, followed by gradual transfer to 100% glycerol for the final clearing and storage [59].

**Fig 6:**
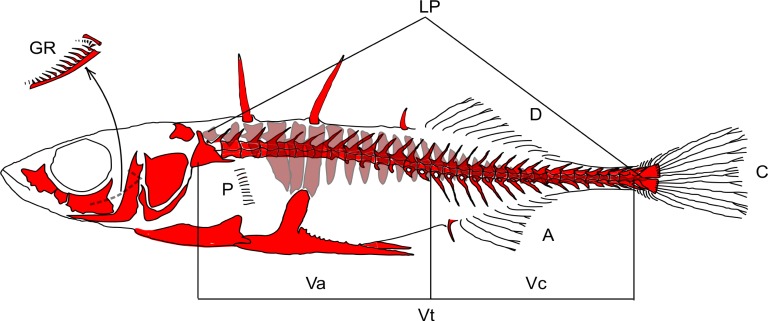
Meristic characters of *Gasterosteus aculeatus*. A–number of anal fin rays, C–caudal fin rays, D–dorsal fin rays, P–pectoral fin rays (clipped fin shown), LP–total number of lateral plates, GR–number of gill rakers on the lower and upper branches of the first branchial arch, Vt–number of total vertebrae, Va–abdominal vertebrae, and Vc–caudal vertebrae.

In addition to analysis of lateral plates development, we also studied definitive (adult) number of LP as well as other meristic traits, including number of anal (A), caudal (C), dorsal (D), and pectoral (P) fin rays; total number of lateral plates (LP); number of gill rakers on the lower and upper branches of the first branchial arch (GR); number of total (Vt), abdominal (Va), and caudal (Vc) vertebrae (Fig 6.). Number of pectoral rays, lateral plates and gill rakers were counted on both sides and given as averages. Counts of caudal vertebrae included all vertebrae having a normally developed haemal spine [25]. All counts were done by one person, at least twice, under a binocular microscope (Micromed MC-2-ZOOM) in order to minimize errors. Total length (TL) varied between 19.6–54.0 mm and 24.8–42.2 mm for FF and MF fish, respectively. Although some of the examined individuals were smaller than 28 mm in TL (the size at which three-spined stickleback attain an adult phenotype [60]), their meristic measurements did not differ from those of the other fish within the same group, and were hence included in the analyses.

### Thyroid hormone assay

Thyroid hormone (T_3_) levels were analyzed both in wild adult fish (only females with eggs) and experimental juveniles. Blood samples were collected from wild-caught females sampled at the same locations as the parents used in the crosses described above. Blood from several females (4–13 individuals) was pooled for each T_3_-analysis. In total, three pooled blood samples of the FF (from a total of 19 individuals) and six pooled blood samples of the MF (from a total of 37 individuals) were analyzed. Blood was collected into sterile tubes. Plasma was centrifuged (5000 g for 30 min) and frozen at -20°C until assayed. The whole bodies of experimental fish (juveniles, 61 days post fertilization–dpf) were homogenized with preliminary freezing (-20°C). The hormone was extracted from whole-body homogenates in buffer (0.1 M phosphate buffered saline pH 7.4, 0.1% Triton X-100, 1 mM propylthiouracil–Sigma Aldrich). Samples were diluted 1:1 with buffer. Homogenates were centrifuged (10000 g for 30 min) and supernatants were analyzed for T_3_ concentration. Plasma T_3_ concentrations and whole body T_3_ concentrations were assessed by enzyme-linked immunosorbent assay (ELISA) using commercial kits (tT3, Monobind Inc., CA, USA) for total T_3_. The sensitivity of the T_3_ assay was 0.04 ng/ml according to the manufacturer’s instructions. Results were read on a photometer Stat Fax 303 Plus (Awareness Technology Inc., FL, USA) at 450 nm wavelength against 630 nm for the blank solution. All samples were analyzed in duplicate.

### Statistical analysis

Statistical analyses of the data was performed using Statistica® software (Statsoft Inc., ver. 8.0). All data were tested for normality of variance using the Kolmogorov-Smirnov one-sample test and the Shapiro-Wilk W test. Due to small sample sizes and non-normal distributions, non-parametric Mann-Whitney U-test and Kruskal-Wallis tests were used to compare differences among wild populations and experimental groups (Kruskal-Wallis test was followed by the Dunn’s *post hoc* test). Results were considered significant when p < 0.05.

All original data are given in S1, S2, S3 and S4 Tables.

## Supporting information

S1 Fig Additional bony plates detected in wild-caught sticklebacks from the Russian coast of the Gulf of Finland (population code PET. See "Sample collection" in DeFaveri & Merilä [48] for further details on collection).
(DOCX)

S2 Fig PCR genotyping of MF and FF parents using endogenous *Eda* locus polymorphism shows that MF parents are either homozygous for the 218-bp fully-plated allele (lanes 2–9) or heterozygous for the 218-bp allele and the 150-bp low-plated allele (lane 10). In contrast, all FF parents were homozygous for the 150-bp low-plated allele (lanes 11–14, 16–19).
(DOCX)

S1 Table T_3_ concentrations in the wild caught three-spined sticklebacks and in the laboratory-raised three-spined sticklebacks. (XLS)

S2 Table Values of meristic traits in different experimental groups for MF fish. (XLS)

S3 Table Values of meristic traits in different experimental groups for FF fish. (XLS)

S4 Table Development of lateral pates in different experimental groups for MF fish. (XLS)
