# Supplementary material for: Heterochronic development of lateral plates in the three-spined stickleback induced by thyroid hormone level alterations
Source: PLoS One. 2018 Mar 9;13(3):e0194040. doi: 10.1371/journal.pone.0194040 (PMC5844557; doi:10.1371/journal.pone.0194040)
Supplement: S1 Fig — See "Sample collection" in DeFaveri & Merilä [48] for further details on collection). (DOCX) [file pone.0194040.s001.docx]

**Supplementary Information**

**S1 Fig**


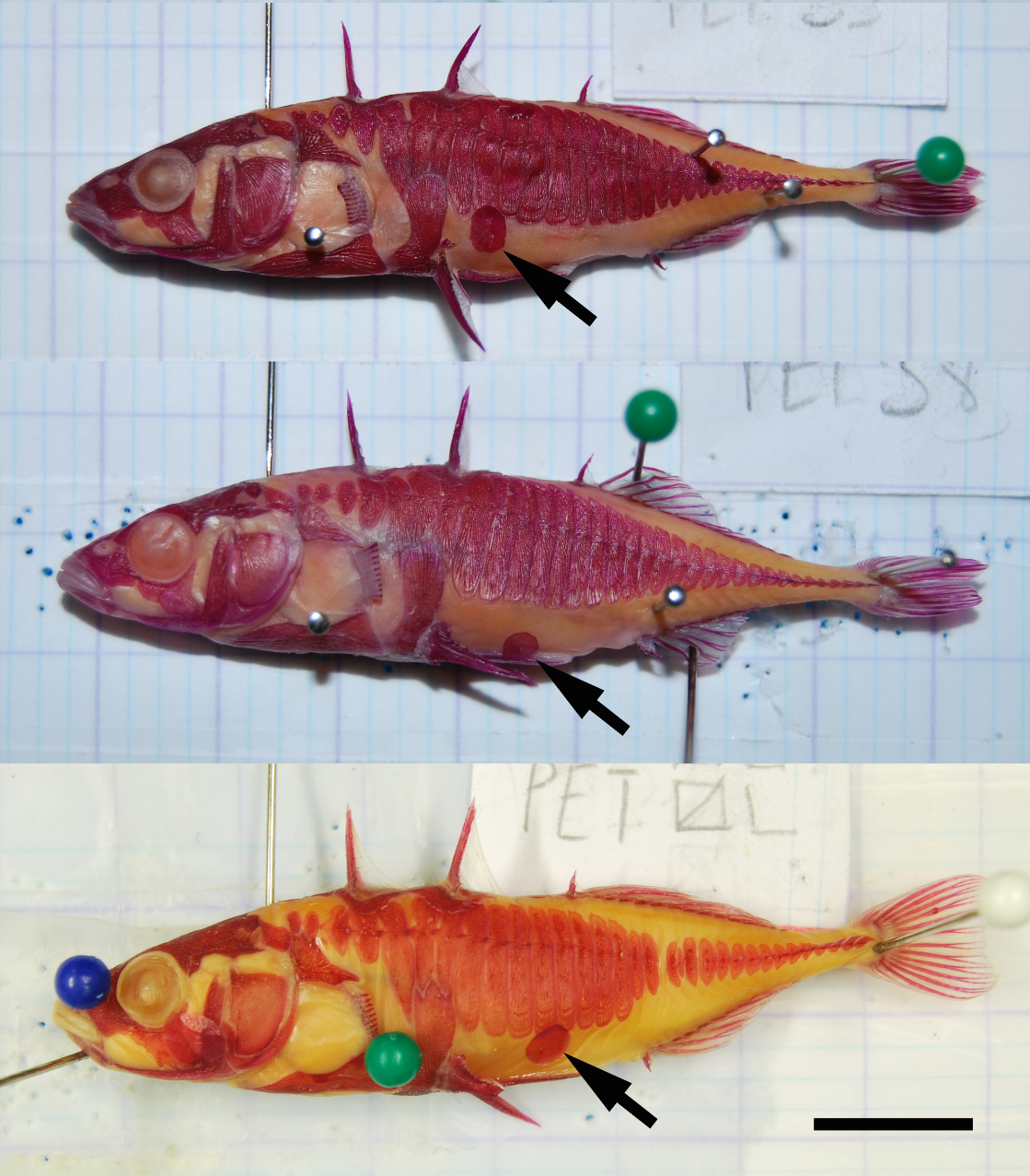


S1 Fig. Additional bony plates detected in wild-caught sticklebacks from the Russian coast of the Gulf of Finland (population code PET. See "Sample collection" in DeFaveri and Merilä [48] for further details on collection).
