# Supplementary material for: Heterochronic development of lateral plates in the three-spined stickleback induced by thyroid hormone level alterations
Source: PLoS One. 2018 Mar 9;13(3):e0194040. doi: 10.1371/journal.pone.0194040 (PMC5844557; doi:10.1371/journal.pone.0194040)
Supplement: S2 Fig — In contrast, all FF parents were homozygous for the 150-bp low-plated allele (lanes 11–14, 16–19). (DOCX) [file pone.0194040.s002.docx]

**Supplementary Information**

**S2 Fig**


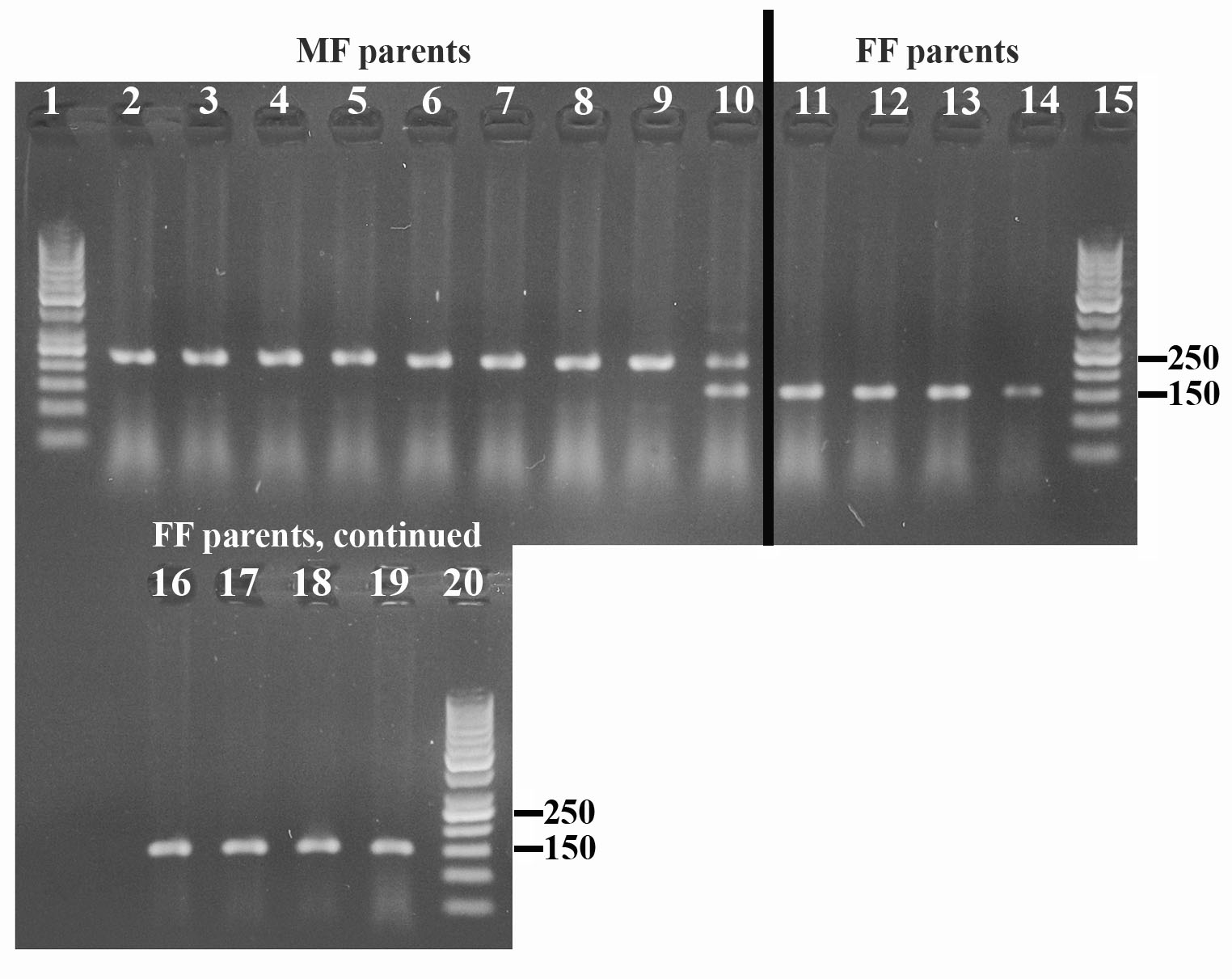


S2 Fig. PCR genotyping of MF and FF parents using endogenous *Eda* locus polymorphism shows that MF parents are either homozygous for the 218-bp fully-plated allele (lanes 2-9) or heterozygous for the 218-bp allele and the 150-bp low-plated allele (lane 10). In contrast, all FF parents were homozygous for the 150-bp low-plated allele (lanes 11-14, 16-19).
